# Economic burden of zoonotic and infectious diseases on livestock farmers: a narrative review

**DOI:** 10.1186/s41043-025-00913-3

**Published:** 2025-05-15

**Authors:** Bibin Bose, S. Siva Kumar

**Affiliations:** Department of Social Sciences, School of Social Sciences and Languages, Vellore Institute of Technology, Vellore, Tamil Nadu 632014 India

**Keywords:** Economic impact, Zoonoses, Mastitis, COVID-19, Livestock farmers

## Abstract

**Background:**

Zoonoses significantly impact human health and agricultural productivity, particularly affecting livestock farmers. In this review, the primary objective was to understand the economic impact of both zoonotic and potential zoonotic diseases.

**Methods:**

This narrative review synthesises literature from SCOPUS, Web of Science, PUBMED, and Reports, covering articles published between 1970 and 2024. Inclusion criteria focused on articles discussing economic losses due to zoonotic diseases in livestock, while exclusion criteria eliminated non-peer-reviewed works and studies not in English.

**Results:**

A total of 37 articles were analysed, revealing substantial economic impacts from various zoonotic diseases. The study uncovers a dramatic decrease in milk consumption, with some areas experiencing a reduction of up to 64 per cent, causing financial hardship for dairy farmers. Moreover, animal-to-human transmissible diseases like bovine tuberculosis, Rift Valley Fever and mastitis result in significant economic setbacks, especially in developing countries.

**Conclusion:**

Addressing economic challenges caused by zoonotic and potential diseases is vital for dairy sector sustainability, particularly in developing nations like India. The study emphasises the need for collaborative efforts from stakeholders, including government officials and researchers. It underlines key challenges and compares economic contexts between countries, advocating increased livestock farmers’ awareness of these diseases, improved farming techniques, and training programmes to alleviate the problem.

## Introduction

Zoonoses are diseases transmitted between humans and animals [1, 2] that pose significant threats to human health and impose severe economic burdens on livestock farmers [3–5]. Over one billion cases of illness and millions of fatalities have been attributed to zoonoses, which account for approximately 60 per cent of all emerging infectious diseases (EIDs) diagnosed globally. Notably, among the 30 new human pathogens identified over the past 30 years, 75 per cent are of animal origin [6]. Given the ongoing emergence and reemergence of various zoonotic diseases worldwide, climate change [7], intensive farming practices [8–10], and inadequate biosecurity measures [11] contribute to the spread of the disease. This underscores the critical potential that these diseases impose on mankind, especially in livestock farmers.

Considering the history of the economic burden, the Plague in India in 1994 was estimated to be between USD 600 million and USD 2 billion, whereas the Highly Pathogenic Avian Influenza (HPAI) pandemic in Asia from 2004 to 2009 resulted in a loss of USD 10 billion. The Nipah virus encephalitis outbreak in Malaysia from 1998 to 1999 resulted in an estimated USD 617 million in damage, and Severe Acute Respiratory Syndrome (SARS) outbreaks in China, Taiwan, Hong Kong, and Singapore cost USD 13 billion [12]. Furthermore, the impact of zoonotic diseases extends beyond health concerns, significantly diminishing animal productivity and increasing veterinary costs, thereby creating a ripple effect of economic strain on farmers [13, 14].

Farmers face numerous challenges in the dairy industry, including low production levels, limited access to artificial insemination, low prices, and restricted credit availability [15–17]. Furthermore, zoonotic outbreaks can disrupt supply chains, reduce the demand for animal products, and threaten food security, particularly in developing countries where livestock farmers often lack adequate healthcare facilities, education, and awareness of these diseases [18–20]. The rising treatment costs and lost market opportunities exacerbate these challenges. Understanding the economic burden of these diseases is crucial to support livestock farmers. Thus, this review aims to explore the narrative of the economic impact of zoonotic and potential diseases globally, bridging the gap by providing a more concrete understanding of the burden of zoonotic diseases on livestock farmers.

## Methods

### Search strategy and data extraction

The literature search for this review was taken from SCOPUS (https://www.scopus.com/home.uri), Web of Science (www.webofscience.com), PUBMED (https://pubmed.ncbi.nlm.nih.gov/) and Google Scholar databases using Boolean operations like “AND” and “OR”. The following keyword search was conducted to extract the articles: “Zoonotic Diseases” OR “Zoonoses” AND “Economic Impact” OR “Economic Loss” AND “Livestock Farmers” OR “Dairy Farmers”. The review covered articles from 1970 to 2024. Apart from this, government reports were also considered from the FAO website (https://openknowledge.fao.org/home).

### Inclusion & exclusion criteria

The following inclusion criteria were used to extract the articles for the review: the articles are either original or review articles which deal with economic loss caused by zoonotic and potential zoonotic diseases, the articles were published between the beginning of 1970 to the end of 2024, the articles are published in the English language. In addition to the above, the review incorporated all the articles published worldwide; including on the type of paper [e.g. original research, review, Book Chapters]. The following exclusion criteria were used, Articles that do not explain the socioeconomic impact caused. Conference Proceedings were not considered as the abstracts did not contain adequate information, No English language articles, and articles published after 30th December 2024 were not incorporated into the study.

### Screening and selection

The reviewer [B.B.] independently reviewed the titles and abstracts from different databases against the set eligibility criteria, and the same was cross-verified by the reviewer [S.S.K.]. An almost perfect agreement was found between the reviewers with a Cohen’s Kappa value of 0.836. All the identified articles were entered into Biblioshiny for the elimination of duplicates from the databases. At this stage, justifications for exclusion were recorded. The two reviewers resolved any differences or disagreements in the selection process through dialogue and mutual agreements.

### Review approach and data analysis

A narrative review approach was adopted to synthesise the evidence on the economic burden of zoonotic disease. This narrative review utilises a qualitative method to examine current data sourced from articles, government reports, and epidemiological studies on zoonotic and infectious diseases in livestock. Subsequently, the analysed reviews are thematically organised and interpreted to emphasise the current direction of research on the economic impact of these diseases.

### Data items

The literature review compiled data on the location of the study, specific infectious or zoonotic diseases examined (such as mastitis, brucellosis, bTB, RVF, AMR), type of study conducted, and anticipated economic losses. Where applicable, additional factors, such as public health impacts and disease management strategies (including vaccination, monitoring, or test-and-slaughter methods) were also documented. These elements were selected to enable thematic and regional comparisons of the economic impacts on livestock producers.

## Results

### Study selection

**Fig. 1:**
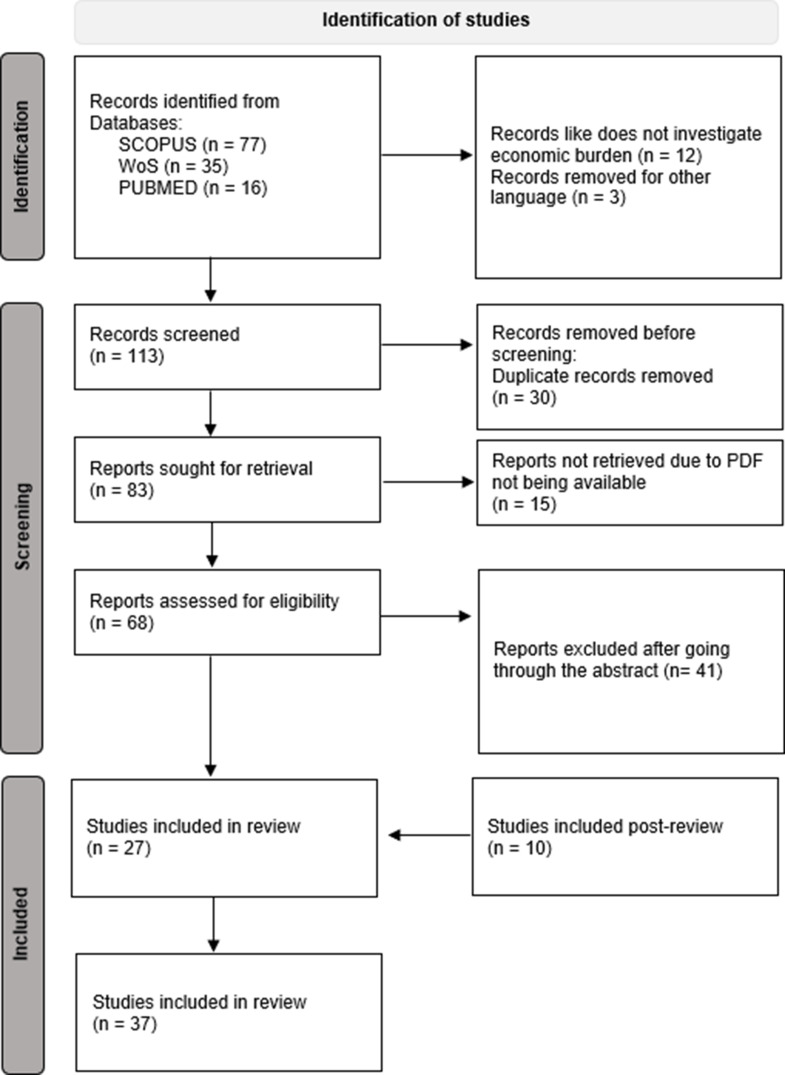
Shows PRISMA flow diagram of literature search

An extensive literature review was conducted using three distinct databases, yielding 128 relevant publications (Fig. 1). Following the elimination of duplicates and the initial screening, 123 documents were selected for further examination. Owing to the unavailability of full texts, 15 documents were excluded from the study, resulting in 78 articles being selected for retrieval. After a thorough abstract review, 41 of these 78 articles were subsequently removed, leaving 27 articles for comprehensive full-text evaluation. Post-revision 10 articles were added, making a final of 37 articles for full-text analysis. The entire study is summarised into 3 tables to provide a clearer understanding of the geographical area. Table 1 provides the holistic picture of studies conducted in the Global, European, and North American context, while Table 2 covers studies in Asian Countries and finally Table 3 summarises research in African countries and Table 4 deals with studies on other parts of the world. These tables serve as a framework for the detailed thematic analysis.

**Table 1:** Studies conducted in global, USA, and European countries context

Country/Region	Disease(s)	Study Type	Reference
Global	Zoonotic Diseases	Review	Hinchliffe et al. [21]
Global	Mastitis	Review	Gayathri et al. [22]
Global	Zoonotic Spillover	Review	Astbury et al. [23]
Global	Mastitis	Review	Soundhararajan et al. [24]
Global	Mastitis	Review	Kour et al. [25]
Global	Viral Zoonoses	Economic Modeling	Bernstein et al. [26]
Global	COVID-19	Commentary	Abhijit et al. [27]
Global	Antimicrobial Resistance	Projection	Vijay et al. [28]
Global	Mastitis	Review	Kratochvilova et al. [29]
Global	Brucellosis	Cost-effectiveness	Kiiza et al. [30]
Global	Mastitis	Review	Halasa et al. [31]
USA	COVID-19	Survey	Murakami et al. [32]
USA	Mastitis	Economic Estimate	Oyelami et al. [33]
USA	COVID-19	Case Study	Seidel et al. [34]
Turkey	Zoonotic Diseases	Economic Estimate	Ari et al. [35]

**Table 2:** Studies conducted in Asian countries context

Country/Region	Disease(s)	Study Type	Reference
Pakistan	COVID-19	Survey	Ul Ain et al. [36]
India	COVID-19	Cost-Analysis	Arora et al. [37]
India	COVID-19	Economic Survey	Thejesh et al. [38]
India	COVID-19	Market Study	Haritha et al. [39]
Bangladesh	COVID-19	Sector Impact	Rahman & Chandra Das [40]
India	COVID-19	Survey	Bhandari et al. [41]
India	Mastitis	Lab Analysis	Goncalves et al. [42]
India [Punjab]	Tick-borne Diseases	Economic Modelling	Hussain et al. [43]
India [Kerala]	AMR & Mastitis	Survey	Lejaniya et al. [44]
India [North]	Calf Scour	Field Survey	Brar et al. [45]
India [Kerala]	Mastitis	Cost Estimation	Dinesh et al. [46]
India [Punjab]	Zoonotic Disease [General]	KAP	Singh et al. [20]

**Table 3:** Studies conducted in African countries context

Country/Region	Disease(s)	Study Type	Reference
Kenya	RVF	Cost Estimation	Tinto et al. [47]
Ethiopia	Mastitis	Epidemiological	Abebe et al. [48]
Nigeria	Fasciolosis	Economic Modelling	Odeniran et al. [49]
Ethiopia	Bovine TB	Review	Kemal et al. [50]
Egypt	Brucellosis	Burden Estimate	FAO [51]
Egypt	Avian Influenza	Burden Estimate	FAO [51]
Kenya	Bovine TB	Burden Estimate	FAO [52]
Kenya	Brucellosis	Burden Estimate	FAO [52]
Kenya	Salmonellosis	Burden Estimate	FAO [52]
Nigeria	Bovine TB	Burden Estimate	FAO [53]
Nigeria	Brucellosis	Burden Estimate	FAO [53]
Nigeria	Salmonellosis	Burden Estimate	FAO [53]
Nigeria	Avian Influenza	Burden Estimate	FAO [53]
Uganda	Bovine TB	Burden Estimate	FAO [54]
Uganda	Brucellosis	Burden Estimate	FAO [54]
Uganda	Salmonellosis	Burden Estimate	FAO [54]
Uganda	Avian Influenza	Burden Estimate	FAO [54]

**Table 4:** Studies conducted on other parts

Countries/Region	Disease(s)	Study Type	Reference
LMICs	Milk-Borne Disease	Cross-Sectional Study	Prakashbabu et al. [55]
New Zealand	Leptospirosis	Economic Modelling	Sanhueza et al. [56]

### Economic impact of the COVID-19 pandemic on dairy farming

The COVID-19 pandemic has had a profound economic impact on the dairy sector in various regions. In Pakistan, milk demand has dropped by 64 per cent in Bahawalpur, leading to significant financial losses for farmers who had to sell animals and lay off employees due to disrupted veterinary services [36]. Similarly, dairy farmers face income reductions of up to one-third from processed products such as ghee and butter, compounded by labour shortages and increased production costs [37]. In Pennsylvania, 42 per cent of farmers reported revenue losses, whereas vegetable growers fared better than livestock producers, highlighting the adaptability of some sectors through direct-to-consumer sales [32, 34]. In Karnataka, farmers experience drastic reductions in net returns due to decreased milk yields and rising feed costs, particularly affecting crossbred cattle farmers [38]. These farmers experienced an average loss of INR 7,175 per milch animal, primarily because of decreased milk prices and increased production costs exacerbated by supply chain disruptions. The study revealed that farmers selling directly to consumers suffered the most, as consumer preferences shifted towards packaged milk during the lockdown [39]. The overall economic fallout is severe, with the dairy sector in Bangladesh experiencing daily losses of 570 million BDT [40]. The economic repercussions of the COVID-19 lockdown led to a decrease in feed and fodder availability by 11 per cent, a rise in production costs by 6 per cent, and a drop in milk prices by 5.6 per cent, contributing to an estimated financial loss of INR 4,000 per milch animal [41]. Likewise, a meta-analysis of the economic impact of COVID-19 on dairy farmers identified negative impacts such as limited availability of dairy inputs and reduced farm gate prices as primary concerns, leading to severe financial strain for producers. The analysis notes that many farmers were forced to dump unsold milk because of market access issues, while dairy cooperatives experienced a 40 per cent drop in demand, highlighting the disruptions caused by the pandemic [27].

### Impact from other zoonotic diseases

Zoonotic diseases pose a significant economic challenge to livestock farmers. For instance, zoonotic risks in livestock agriculture are significantly influenced by social, economic, and political factors, and full appreciation of these factors is required for accurate risk assessments [21]. In the case of the Rift Valley Fever (RVF) outbreak in West Africa, which caused an economic impact on a multitude, such as at the individual level, livestock mortality and abortions cause substantial monetary losses for producers. At the community scale, RVF disrupts livestock marketing chains, reducing rural livelihoods and agricultural product value. Globally, RVF affects urbanised areas, leading to reduced economic activity and unemployment. It also results in trade bans, complicating international livestock commerce, and explains that in Kenya, RVF estimated a loss of USD 32 million [47]. In the case of brucellosis control interventions, vaccination alone is generally cost-effective, with benefit-cost ratios [BCRs] ranging from 3.2 to 21.3, while test-and-slaughter methods are not cost-effective, showing BCRs from − 1.2 to 0.6. The combination of both strategies has yielded mixed results [30]. In another estimate, the annual gross national income (GNI) loss from viral zoonoses was USD 212 billion and the median cost of primary prevention was approximately USD 20 billion, which represents ~ 1/20 of the low-end annualised value of lives lost to emerging viral zoonoses and < 1 / 10 of the annualised economic losses [26]. Similarly, in Turkey, a total economic loss of USD 813 million was estimated in terms of both human and animal origin [35]. In Tick-borne diseases (TBDs) result in annual losses between USD 13.9 to 18.7 billion globally, with tick infestations in Punjab affecting livestock productivity [43]. In Nigeria, bovine fasciolosis leads to annual losses of approximately USD 26.02 million annually, threatening food security and small-scale farmers’ livelihoods [49]. Leptospirosis in New Zealand is associated with an estimated annual cost of USD 18.80 million due to lost productivity and treatment expenses [56]. Furthermore, zoonotic diseases in Punjab, India, have led to decreased livestock productivity and increased healthcare costs, emphasising the need for targeted education on disease prevention [20]. Bovine tuberculosis (bTB) significantly impacts livestock production, with high prevalence rates leading to reduced milk production and increased veterinary costs [50]. In Egypt, the total burden of brucellosis amounted to about USD PPP 367 million USD in 2016, and the total burden of HPAI in Egypt amounted to about 3.9 million USD PPP in 2016 [51]. In Kenya, total burden of Bovine TB is estimated to be 512.1 million USD, the total burden of Brucellosis is estimated to be 4305.5 million USD, and the total burden of Salmonellosis is estimated to be 1061.2 million USD [52]. Similarly, in Nigeria, the total burden of Bovine TB is estimated to be 2994.6 million USD, the total burden of brucellosis is estimated to be 1539.2 million USD, the total burden of Salmonellosis is estimated to be 3677.8 million USD and the total burden of Highly Pathogenic Avian Influenza is estimated to be 1066.7 million USD [53]. In Uganda, the total burden of Bovine TB is estimated to be 246.8 million USD, the total burden of brucellosis is estimated to be 783.2 million USD, the total burden of Salmonellosis is estimated to be 103.4 million USD and the total burden of Highly Pathogenic Avian Influenza is estimated to be 8.9 million USD [54]. The economic implications of calf scour outbreaks in the dairy industry have led to a high mortality rate of 43 per cent among affected calves. This study emphasises the devastating impact of concurrent infections caused by pathogens, such as *Cryptosporidium Parvum*, *Clostridium Perfringens*, and *Salmonella Spp.* High mortality not only threatens farmers’ livelihoods but also disrupts milk production, leading to broader economic repercussions for the dairy sector [45]. Among the 111 policies evaluated for preventing zoonotic spillover that were adopted in different sectors, such as industries, reports underlined the relevance of surveillance data in both guiding preventative efforts and facilitating policy assessment, as well as the importance of industry and private sector players in implementing many of these programs. They discovered that the majority of existing policy evaluations focus on ‘downstream’ determinants; further research could focus on evaluating policies targeting ‘upstream’ determinants of zoonotic spillovers, such as land use change, as well as policies affecting infection intensity and pathogen shedding in animal populations, such as those aimed at animal welfare [23].

### Economic burden of mastitis: a potential zoonosis

Mastitis, particularly when caused by pathogens such as *Staphylococcus aureus*,* Escherichia coli*, and *Streptococcus agalactiae*, are increasingly identified as a potential zoonotic concern, especially posing a great threat when consumed raw or improperly processed milk [57, 58]. Multiple studies documented the multidrug-resistant strains in milk from mastitic cows, underscoring the risk of cross-species transmission and the importance of stringent milk hygiene and pasteurization practices to mitigate zoonotic threats [59, 60]. Mastitis remains one of the most economically significant diseases in dairy farming worldwide, with global losses estimated at 20–30 billion USD annually [22]. It adversely affects both the quality and quantity of milk, particularly in countries like India, where subclinical and clinical mastitis lead to decreased milk quality and quantity, significantly affecting farmers’ finances [42]. It is reported that subclinical mastitis could lead to production losses accounting for 70–80 per cent of the total costs associated with the disease [25].

In Ethiopia, an high incidence rate of 83.72 cases per 100 cow-years highlighted the rapid spread of clinical mastitis, leading to substantial economic repercussions [48]. The significant economic impact of bovine mastitis on dairy farming is due to decreased milk production and increased veterinary costs. It discusses the multifaceted challenges posed by this disease, which is primarily caused by pathogens, such as *Staphylococcus aureus* and *Escherichia coli*. and these causative agents continuously challenges in the effective management and control of the disease.

Despite existing control measures, the limitations of existing vaccines and the urgent need for innovative approaches to effectively manage mastitis are of high priority [29]. In United States alone, the economic losses due to mastitis are estimated at USD 2 billion annually, necessitating effective management strategies and the global scale of the disease [33]. A comprehensive review analysed the economic impact of bovine mastitis on dairy farming, estimating losses per cow ranging from EUR 22 to EUR 287 annually highlighting the variability in cost estimates across different research methodologies and regions, making it difficult to draw definitive conclusions. This underscores the importance of effective mastitis management strategies that can yield significant economic benefits, with some studies reporting favourable benefit-to-cost ratios. The need for more recent and comprehensive research to address gaps in our understanding of the economics of mastitis has also been emphasised [31].

**Fig. 2:**
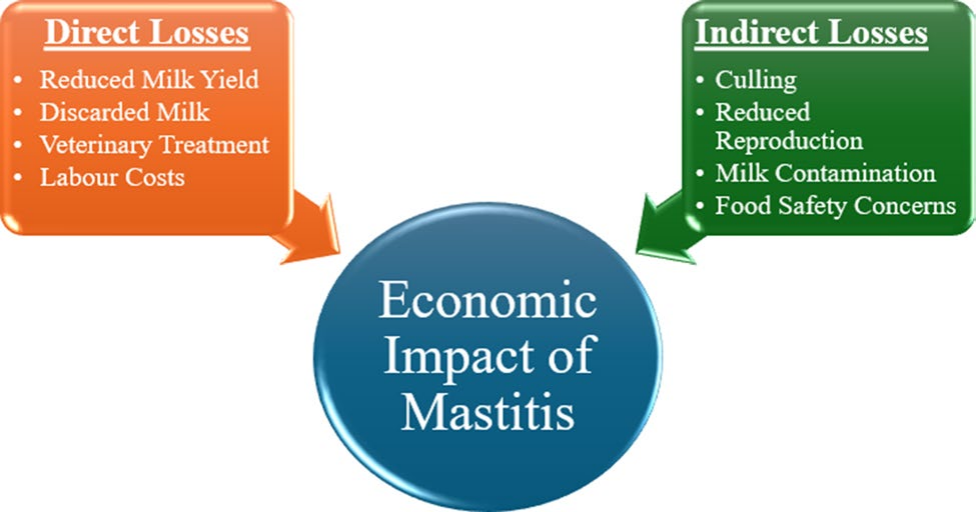
Mastitis-linked economic loss

Thus, in a nutshell, the significant effects of mastitis can be understood in terms of both direct and indirect costs. Mastitis causes inflammation in the mammary glands of dairy animals, leading to decreased milk production and quality. These are depicted in the chart (Fig. 2) as the main direct losses, which include discarded milk, veterinary treatment costs, and extra labour for handling infected animals. Additionally, there are indirect losses such as early culling, reduced reproductive efficiency, and a long-term drop in herd productivity, all of which greatly diminish farmers’ income. This illustrates a complex economic burden, particularly for small-scale farmers.

### Antimicrobial resistance: impact on livestock economics

Antimicrobial resistance (AMR) is a growing threat to both animal and human health, posing a critical threat to the veterinary sector, resulting from the overuse and misuse of antibiotics in livestock production. The misuse of antibiotics in livestock is a significant concern, as it exacerbates the emergence of resistant strains [46]. Alarmingly AMRis projected to contribute to 10 million deaths and USD 100 trillion in global economic losses by 2050, particularly in developing countries [28].

While antibiotics are essential for managing bacterial infections such as mastitis, indiscriminate usage contributes to the development of multidrug-resistant strains, there by complicates treatment and increases veterinary costs [24]. For instance, Mastitis alone imposes a significant economic burden on dairy farmers, with losses of up to 70 per cent in milk yield and substantial treatment costs. In India, the annual economic loss due to udder infections is estimated at INR 0.6053.21 crores, reflecting the urgent need for effective management strategies.

The economic impact of AMR is multifaceted, encompassing prolonged illness in animals, higher veterinary costs, reduced productivity, and restrictions on trade. In Senegal, milk-borne diseases highlight the economic impact of unsafe food practices, with cultural beliefs complicating efforts to improve food safety, linking that AMR can affect both human-animal health and public confidence [55]. Additionally, the prevalence of subclinical mastitis, affecting 25 per cent of the surveyed cows, further illustrates the challenges posed by antibiotic resistance and its economic implications [44].

**Fig. 3:**
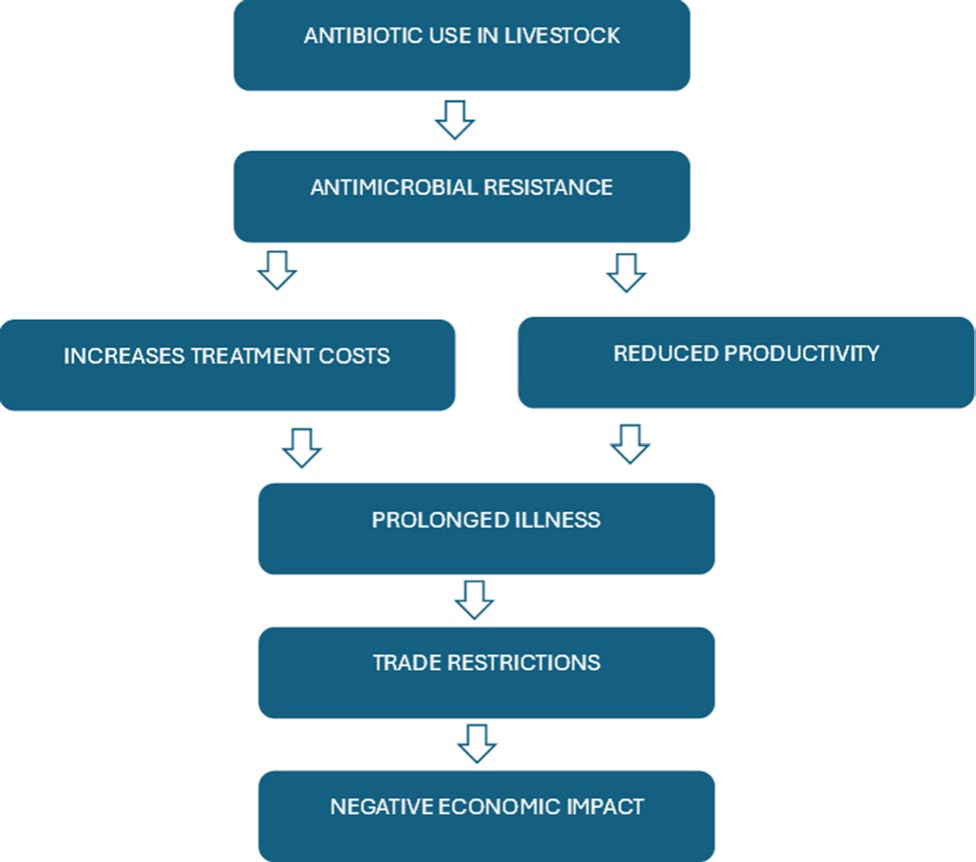
Economic implications of AMR in livestock farming

Overall, the economic burden of AMR (Fig. 3) explains the complex pathways through which AMR emerges and leads to be a significant economic consequence in livestock farming as explained by the past studies and underscores the urgent need for improved management practices, responsible antibiotic stewardship, sustainable livestock health management practice and awareness among farmers to mitigate these challenges effectively.

## Discussion

The economic impacts of the COVID-19 pandemic and zoonotic diseases on dairy farming are multifaceted, revealing significant disparities between developed and developing nations. While the data presented elucidate the severe financial strain on farmers globally, a critical assessment of the study limitations and contextual challenges is imperative to comprehensively understand the implications of these findings.

One of the primary challenges in mitigating the economic impacts of diseases, such as mastitis and zoonotic infections, is the financial constraints faced by farmers. Many small-scale dairy farmers operate on thin margins in developing countries, such as India and Pakistan, making it difficult to invest in advanced management practices or veterinary care [42]. The high costs associated with vaccination, treatment, and compliance with food safety standards can be prohibitive. Moreover, a lack of awareness regarding disease prevention and management strategies exacerbates this situation. Many farmers may not recognise signs of mastitis or zoonotic diseases, leading to delayed interventions and greater economic losses [29].

Additionally, infrastructure for veterinary services is often lacking in rural areas, which can hinder timely access to treatment and resources. This situation contrasts sharply with that of developed nations, where farmers typically have better access to veterinary care and education about disease management. For instance, in the U.S. and EU, farmers benefit from robust extension services that provide training on best practices, thereby reducing the incidence of diseases, such as mastitis, and enhancing overall productivity [33].

The economic burden of diseases in dairy farming varies significantly across different geographic regions. In developed countries such as the U.S. and New Zealand, economic losses due to mastitis are estimated at approximately USD 2 billion annually [33]. Farmers in these regions often have better access to technology and resources, allowing for more effective disease management strategies. Stringent regulations and strong market demand for high-quality dairy products further incentivise farmers to adopt best practices, resulting in lower disease prevalence and economic losses. In contrast, developing nations face a higher disease burden owing to a combination of factors, including inadequate veterinary services, limited access to quality feed, and poor sanitation practices. For example, the economic impact of bovine mastitis in India is compounded by the presence of multidrug-resistant pathogens, which complicate treatment efforts and increase veterinary costs [24]. Gonçalves and others highlighted that in India, subclinical and clinical mastitis significantly affect milk quality and quantity, leading to substantial financial losses for farmers who already operate on precarious margins [61]. Moreover, zoonotic diseases, such as RVF, present a unique challenge in African countries, where outbreaks can lead to massive economic losses and disrupt livestock marketing chains [47]. The economic loss from RVF in Kenya alone is estimated at USD 32 million, showing the substantial impact of zoonotic diseases on rural livelihoods [47]. In these regions, the interplay of social, economic, and political factors further complicates the risk assessment and mitigation strategies [21].

Brucellosis significantly affects smallholder farmers by reducing milk yield and causing reproductive issues [30, 51]. Additionally, it poses a major public health risk in areas where unpasteurised dairy products are frequently consumed. In humans, chronic brucellosis can result in prolonged disability, exacerbating poverty in communities dependent on livestock [55]. Similarly, bTB not only lowers productivity, but also creates occupational risks for farm workers and slaughterhouse staff, necessitating more comprehensive health strategies [50, 53].

Notably, the methods of managing brucellosis and bTB differ significantly. In the EU, developed countries have adopted stringent test and slaughter initiatives supported by robust compensation and monitoring systems. These efforts have enabled several EU nations to attain an official disease-free status [52]. In contrast, many developing nations lack the financial resources or institutional frameworks to implement similar strategies. For example, India utilises Brucella abortus S19 and RB51 vaccines as control measures, but coverage is inconsistent and follow-up monitoring is often inadequate [30]. In numerous regions of Africa and Asia, tuberculosis control is disjointed because of the lack of diagnostic facilities and regular screening [50, 54].

Cultural norms contribute to the ongoing presence of the disease. Activities like consuming unpasteurized milk and maintaining close contact between humans and livestock facilitate the spread of zoonotic diseases [55]. As a result, families involved in livestock farming in low-income areas find themselves trapped in a cycle where illness reduces their earnings potential, and financial instability hinders their ability to invest in preventive measures [20, 53].

Examining regional differences, for example, reveals that losses due to mastitis vary significantly between developed countries such as the United States, where management practices are advanced yet costs remain substantial, and nations such as India and Ethiopia, where controlling the disease is difficult and smallholder farmers face severe economic challenges [33, 42, 48]. Likewise, the effects of brucellosis are more severe in low- and middle-income countries, where control measures are insufficient, and public health risks are closely linked to reliance on livestock [30, 51, 55]. This highlights that the economic impact of these diseases shows considerable regional variation, shaped by complex factors, such as disease prevalence, animal husbandry practices, access to veterinary care, and policy enforcement.

These findings highlight the importance of addressing not only the biological effects of zoonotic diseases but also their connection to socioeconomic conditions. To effectively manage these diseases, strategies must be tailored to specific regions to ensure that they are economically feasible and culturally sensitive [29, 55]. This approach involves enhancing veterinary services, broadening farmer education, and encouraging the reporting of diseases and adherence to regulations [20, 42].

An integrated One Health framework that connects animal, human, and environmental health is increasingly acknowledged as the most feasible path forward [21, 28]. By coordinating resources across different sectors and emphasising early detection and response, this strategy can help mitigate the risk of spillover events and reduce long-term economic impact [28, 51]. However, such cross-sector collaboration requires enhanced policy support, investment in veterinary infrastructure, and capacity building at the local level to ensure sustainability and resilience in communities reliant on livestock [51, 54].

## Conclusion

The economic ramifications of diseases such as mastitis and zoonotic infections are apparent across diverse geographical regions; however, the obstacles encountered by farmers in implementing efficacious management strategies warrant careful consideration. In developing nations, fiscal limitations, insufficient awareness, and inadequate veterinary infrastructure have substantially impeded disease mitigation endeavours. Conversely, agriculturists in developed countries enjoy superior resources, educational opportunities, and market conditions, resulting in diminished disease burden and economic loss.

Addressing the economic challenges posed by diseases in the dairy industry requires a multifaceted approach involving policymakers, researchers, and farmers. Governmental bodies should allocate funds for veterinary services and knowledge dissemination in developing nations to facilitate farmers’ access to timely information and resources for disease management. This approach should encompass subsidised immunisation programs and educational initiatives to elucidate optimal animal health practices. The scientific community must concentrate on devising cost-effective solutions tailored to the needs of smallholder farmers, including innovative diagnostic tools and treatments for conditions such as mastitis and zoonotic infections. Moreover, comparative analyses of disease management strategies across various regions may yield valuable insights into effective methodologies.

Although this study provides an in-depth analysis of the economic challenges that zoonotic and emerging diseases present to livestock farmers, it has some limitations. First, as a narrative review, it does not adhere to a systematic review approach, which could lead to selection bias in the literature included. Second, inconsistency in data availability across different regions and diseases complicates the uniform comparison of economic impacts, particularly in low- and middle-income countries where surveillance and reporting are often inadequate. Third, the lack of uniformity in economic evaluation methods, such as varying cost estimation models, currencies, and timeframes across studies, may limit the generalisability of the results. Furthermore, the absence of detailed data distinguishing between direct and indirect economic losses limits the depth of the economic analysis. Finally, this review might not fully reflect the rapidly changing landscape of emerging diseases, such as the post-pandemic effects of COVID-19, due to delays in publication cycles.

The study once again wants to emphasise the need for subsidised veterinary care and insurance for smallholder farmers, as well as, an urgent need to strengthen the surveillance and reporting system, especially in case of zoonotic and infectious diseases. The research encourages further research on addressing economic burden as well as on the awareness aspect, especially on the recent pandemic from other parts of the developing countries, so that farmers can adopt comprehensive management approaches incorporating regular veterinary examinations, stringent hygiene protocols, and enhanced nutritional regimens to bolster animal health, as well as, a region-specific economic model to understand the impact in different region and a socioeconomic study especially on the indirect cost of these diseases are well in scope for further research. These could be attained by fostering collaboration among these key stakeholders, it is feasible to establish a more resilient dairy sector that mitigates the economic consequences of diseases and promotes sustainable livelihoods for farmers on a global scale.

## Data Availability

No datasets were generated or analysed during the current study.

## Abbreviations

AMR: Antimicrobial Resistance
BCR: Benefit-Cost Ratio
BDT: Bangladeshi Taka
bTB: Bovine Tuberculosis
COVID-19: Coronavirus Disease 2019
EU: European Union
HPAI: Highly Pathogenic Avian Influenza
INR: Indian Rupee
RVF: Rift Valley Fever
TB: Tuberculosis
USD PPP: US dollar Purchasing Power Parity
